# Development of Novel EE/Alginate Polyelectrolyte Complex Nanoparticles for Lysozyme Delivery: Physicochemical Properties and In Vitro Safety

**DOI:** 10.3390/pharmaceutics11030103

**Published:** 2019-03-01

**Authors:** Sabrina Sepúlveda-Rivas, Hans F. Fritz, Camila Valenzuela, Carlos A. Santiviago, Javier O. Morales

**Affiliations:** 1 Department of Pharmaceutical Science and Technology, School of Chemical and Pharmaceutical Sciences, University of Chile, Santiago 8380494, Chile; rivas.riveros@ug.uchile.cl (S.S.-R.); hans.fritz@ug.uchile.cl (H.F.F.); 2 Advanced Center for Chronic Diseases (ACCDiS), Santiago 8380494, Chile; 3 Department of Biochemistry and Molecular Biology, School of Chemical and Pharmaceutical Sciences, University of Chile, Santiago 8380494, Chile; kamo.valenzuela@gmail.com (C.V.); csantiviago@ciq.uchile.cl (C.A.S.)

**Keywords:** coacervation, biologics, complex polyelectrolyte, sustained release, lysozyme, encapsulation, protein delivery, polymeric nanoparticles

## Abstract

The number of biologic drugs has increased in the pharmaceutical industry due to their high therapeutic efficacy and selectivity. As such, safe and biocompatible delivery systems to improve their stability and efficacy are needed. Here, we developed novel cationic polymethacrylate-alginate (EE-alginate) pNPs for the biologic drug model lysozyme (Lys). The impact of variables such as total charge and charge ratios over nanoparticle physicochemical properties as well as their influence over in vitro safety (viability/proliferation and cell morphology) on HeLa cells was investigated. Our results showed that electrostatic interactions between the EE-alginate and lysozyme led to the formation of EE/alginate Lys pNPs with reproducible size, high stability due to their controllable zeta potential, a high association efficiency, and an in vitro sustained Lys release. Selected formulations remained stable for up to one month and Fourier transform-Infrared (FT-IR) showed that the functional groups of different polymers remain identifiable in combined systems, suggesting that Lys secondary structure is retained after pNP synthesis. EE-alginate Lys pNPs at low concentrations are biocompatible, while at high concentrations, they show cytotoxic for HeLa cells, and this effect was found to be dose-dependent. This study highlights the potential of the EE-alginate, a novel polyelectrolyte complex nanoparticle, as an effective and viable nanocarrier for future drug delivery applications.

## 1. Introduction

In recent years, the number of proteins and peptide-like drugs in the pharmaceutical industry has increased due to significant therapeutic efficacy and remarkable selectivity, high specificity, activity, minimal drug–drug interactions, and toxicity [1,2]. These new classes of protein-based drugs are generally referred to as ‘biologics’ and include molecules such as insulin, growth factors, and engineered antibodies [3]. Biological drug products in use nowadays are commonly administered via parenteral. Despite the benefits of the parenteral route, some drawbacks include the problem of uncontrollable and undesirable bio-distribution and the uncontrollable metabolism and elimination of biologics [4], difficulties for initiating, and maintaining an injectable treatment in ambulatory patients, i.e., administration technique or initial rejection to injectable treatments [5], the appearance of adverse effects [6], and problems at the site of injection such as pain, bruise, or itching [7]. 

The oral route of drug administration remains to be the ultimate goal of any new drug therapy; however, it presents difficulties such as: Very limited biologics bioavailability through the oral route, due to their higher molecular weights (>1000 Da) limiting membrane passage; the enzymatic and acid metabolism; extensive exposure across the gastrointestinal tract surface area; and the hepatic first past effect [8]. This has led researchers to accelerate the development of novel delivery systems [1,2] that can maintain the structure, biological activity, and viability of biologics, and that are also non-immunogenic, can release the therapeutic agent predictably over time and erode to metabolites that are either absorbed or excreted [9].

Therefore, numerous efforts are underway to improve the bioavailability of biologics using nanoparticulate delivery systems [10]. Promising delivery systems based on polymeric nanoparticles have attracted significant attention and can be synthesized through various techniques including emulsification, reverse microemulsion, polymerization of biodegradable and non-biodegradable monomers, and coacervation. In particular, coacervation leads to the formation of a matrix system in an aqueous medium, also known as the polyelectrolyte complex (PECs), formed between at least two oppositely charged molecules, one of which can be a polycation or polyanion. Thus, mixing have been extensively investigated for drug delivery [11,12] probably due to the fact that the spontaneous association of the oppositely-charged polymers leads to the formation of PECs through strong and reversible electrostatic links [13]. The properties of the PECs are influenced by the chemical composition of the polymers (their molecular weight, stereochemistry, charge densities, etc.), and also by experimental conditions like the concentrations of the polyelectrolytes prior to mixing, their mixing ratio, ionic strength of the solution, and mixing order among others [14,15].

Polymer-polymer, polymer-drug, polymer-drug-polymer, and polymer-protein complexes are types of PECs that have been frequently studied for drug delivery and biomedical applications [16,17,18,19,20]. Here, we describe the focus on polymer-drug (protein)-polymer based coacervation.

Polyelectrolyte complex nanoparticles obtained by complex coacervation, encapsulate the biologic component in the polymer matrix at a molecular level. They offer greater advantages improving/and altering physicochemical characters like stability, can protect biologics, as well as controlling release and thus pharmacological activity [21,22]. Furthermore, they are widely used due to the better functional properties they offer in comparison to using polyanions and polycations alone [23]. For example, alginate/chitosan, dermatan sulfate/chitosan polyelectrolyte complex, and Eudragit L100-55/chitosan for biologic-loaded nanoparticles have been described for entrapment of hydrophilic polypeptides [24,25,26]. Some of these polymers have been extensively investigated in recent years as effective drug delivery devices for some compounds of interest, such as diclofenac sodium [27], gliclazide [28], and diltiazem hydrochloride [29], demonstrating the potential of these particles to effectively deliver these small molecules to target sites and potentially increase their therapeutic benefit [27,28,29]. Moreover, reported alginate-acrylate drug delivery systems have been developed and their physicochemical characteristics evaluated, highlighting their potential as possible drug carriers [30,31,32].

In the present work, the nanoparticles are prepared by using a cationic polymethacrylate derivative (Eudragit E, EE) and alginate as polyelectrolytes and we selected lysozyme as a biologic drug model. Alginate is an anionic polymer derived from marine brown algae cell walls, consisting of a chain of (1-4)-linked β-d-mannuronic acid and α-l-guluronic acid in different arrangements of residues. Alginate is a natural, biodegradable, and muco-adhesive polymer that does not produce toxicity upon oral administration [33,34]. Alginate is hemocompatible and has shown in vivo degradation, resulting in the absence of accumulation in major organs within the body [35]. EE is a cationic copolymer of 2-dimethylaminoethyl methacrylate, methyl methacrylate, and n-butyl methacrylate (2:1:1), non-biodegradable, nontoxic, and is widely used in coating and film forming [36] applications in the pharmaceutical industry [37,38].

Lysozyme (Lys) is a 14.3 kDa enzyme that damages bacterial cell walls by catalyzing the hydrolysis of β-1,4-linkages between N-acetylmuramic acid and *N*-acetyl-*d*-glucosamine residues present in the peptidoglycan layer surrounding the bacterial cell membrane [39]. Lysozyme is a suitable model protein for its structural and enzymatic properties [40,41]. In fact, several new delivery systems have been loaded with lysozyme as a basic protein model to evaluate the effectiveness of biologics delivery, including heparin-doped porous CaCO_3_ particles [42], Fe nanoparticles (FeNP) [43], and dyed nano-fibrous membranes [44].

In this study, we development a novel polymeric nanoparticle obtained from EE and alginate as a delivery system for the biologic model lysozyme and it was our goal to investigate the effect of formulation variables such as total charges and charge ratios over particle physicochemical properties as well as their influence over in vitro nanoparticle safety. The evidence presented here should facilitate the design of effective delivery systems for the encapsulation, protection, and sustained release of bioactive compounds that have the potential to improve human health.

## 2. Materials and Methods

### 2.1. Materials

Dimethylaminoethyl methacrylate copolymer, Eudragit E (EE), was kindly donated by Evonik industries (Essen, Germany). Low molecular weight sodium alginate (Alg), hen egg white lysozyme, fetal bovine serum (FBS), 1X trypsin–EDTA, penicillin–streptomycin solution and syringe filters 0.22 μm were obtained from Sigma (St. Louis, MO, USA). Double distilled water was used for all the experiments and was produced in house (Milli-Q- Directed; Millipore, SAS- 67120, Molsheim, France). CellTiter 96^®^ AQueous Non-Radioactive Cell Proliferation Assay and CytoTox 96^®^ Non-Radioactive Cytotoxicity Assay were purchased from Promega (Madison, WI, USA). HeLa cells (American Type Culture Collection CCL-2) derived from a cervical carcinoma were used and Amicon Ultra-4 (Ultracel-100k) centrifugal filter devices were obtained from Merck Millipore (Cork, Ireland).

### 2.2. Preparation of Nanoparticles

Polymeric nanoparticles were fabricated by coacervation of aqueous EE (polycation) and aqueous alginate (polyanion) solutions. The polyanion solution was added over the polycation solution under controlled conditions of pH (4.8), at 25 °C, and under vigorous stirring of 600 rpm for 25 min.

pNPs formation is driven by the electrostatic interactions among the cationic and anionic polymers under controlled concentrations and mixing conditions [18].

The amounts and ratios of polymers mixed were determined by quantifying the charge contributions of each polymer based on the mass used and the monomer charge density. The Charge Ratio (CR) was defined as the quotient of positive charges and negative charges, while the sum of positive and negative charges was defined as Total Charges (TC) and were calculated as it follows (Equations (1) and (2)):

(1) $$\mathrm{CR}=\frac{{\mathrm{charge}}_{\mathrm{EE}}\;\left(\mu \mathrm{mol}\right)}{{\mathrm{charge}}_{\mathrm{Alg}}\left(\mu \mathrm{mol}\right)}$$

(2) $$\mathrm{TC}={\mathrm{charge}}_{\mathrm{EE}}\;\left(\mu \mathrm{mol}\right)+{\mathrm{charge}}_{\mathrm{Alg}}\left(\mu \mathrm{mol}\right)$$

where charge_EE_ is the total amount of positive charges contributed by EE (in µmol) and charge_Alg_ is the total amount of negative charges contributed by alginate (in µmol). Several CR and TC combinations were evaluated to find the optimal conditions that would allow nanoparticle formation [16]. The charge ratio was evaluated at 0.5, 1.33, and 10, while the total charge (n+ + n−) at 10, 20, and 30 µmol. EE with a weight average molar mass (*M*_w_) of approximately 135.000 g/mol and low-molecular-weight and low viscosity Alg were dissolved in mili Q water to prepare stock solutions 1% *w*/*v*. Aliquots of these solutions were extracted to account for the required µmol based on the CR and TC desired. The final volume was fixed at 6 mL after polyelectrolyte mixing.

To investigate Lys loading in nanoparticles, aliquots of an aqueous Lys stock solution were added to alginate (Alg) solutions before adding under gentle magnetic stirring to the polycation solution. The amount of Lys was expressed as % *w*/*w* based on the total weight of polymers at play.

### 2.3. NP Physicochemical Characterization

The physicochemical parameters of average pNPs size, size distributions, and polydispersity were determined by dynamic light scattering and zeta potential was determined by micro laser Doppler electrophoresis using a Malvern NanoZS (Malvern Instruments Ltd., Worcestershire, United Kingdom) at 25 °C. The mean pNPs size was determined by calculating the diameter from the cumulative function of NP light scattering intensity.

### 2.4. Scanning Electron Microscopy (SEM)

Selected pNPs formulations were lyophilized and then placed over SEM mounts, previously covered with a carbon adhesive layer. Samples were sputter coated with a gold target to a thickness of 10–12 nm in a high vacuum evaporator (108 Auto, Cressington Scientific Instruments Ltd., Watford, UK). The samples were analyzed by SEM in an Inspect F50 (FEI, Hillsboro, OR, USA).

### 2.5. Fourier Transform Infrared Spectroscopy (FT-IR) Study

FT-IR spectra were obtained on an Interspec 200-X FT-IR spectrometer to investigate the interactions among EE, alginate and lysozyme native, and EE/alginate Lys loaded pNPs. Briefly, 2 mg of powdered samples were gently mixed with KBr (at a 1:10 ratio) to obtain pellets using a hydraulic press. FT-IR spectra were scanned in the range of 4000 and 500 cm^−1^ at a resolution of 4 cm^−1^. All data were collected and extracted using Essential FTIR^®^ Spectroscopy Toolbox software.

### 2.6. Nano Tracking Analysis

A NS300 NanoSight (Malvern Instruments Ltd., Worcestershire, United Kingdom) was used and all samples were diluted 1:100 to an appropriate concentration using filtered, bi-distilled water prior to analysis. Water was evaluated first for any particle content prior to diluting the samples. Each analysis was conducted in triplicate for each sample, resulting in video captures per sample at each flow speed. The scattered light of the particles was captured by a sCMOS camera. All measurements were performed at 25 °C. The mean size values obtained by the NTA software correspond to the arithmetic values calculated with the sizes of all the particles analyzed by the software.

### 2.7. Lysozyme Association Efficiency

The association efficiency (AE%) was determined indirectly by quantifying Lys in supernatants after separation by centrifugation of pNPs and non-encapsulated Lys (16,000 g, 20 °C for 30 min). The following equation was used to determine AE%:

$$\mathrm{AE}\;\left(\%\right)=\frac{\mathrm{encapsulated}\;\mathrm{amount}\;\mathrm{of}\;\mathrm{lysozyme}}{\mathrm{theorical}\;\mathrm{total}\;\mathrm{amount}\;\mathrm{of}\;\mathrm{lysozyme}}\times 100$$

Lys content in pNPs was quantified by a validated reverse phase HPLC (RP-HPLC) method. A known weight of dry solids was dissolved in sufficient pH 7.0 phosphate buffer, and a 500 μL aliquot was filtered and stored in vials for HPLC quantification of lysozyme. A Symmetry 300 C18 column (3.5 μ, 4.6 × 150 mm) was used in a Flexar HPLC (Perkin Elmer, Waltham, MA, USA) for separation. The mobile phase consisted initially of 90% solvent A (water, 5% *v*/*v* acetonitrile and 0.1% *v*/*v* trifluoroacetic acid) and 10% *v*/*v* solvent B (acetonitrile, 5% *v*/*v* water, and 0.085% *v*/*v* trifluoroacetic acid) with a solvent gradient to 60% *v*/*v* solvent B in 16 min and then back to 10% *v*/*v* solvent B in 4 min. The flow rate was set to 1 mL/min, the temperature remained constant at 25 °C, and the injection volume was 50 μL. The limit of quantification was determined at 12.5 μg/mL detecting at 215 nm. The HPLC method was validated with respect to linearity, repeatability, limit of quantification, and detection.

### 2.8. Stability of Unload and EE/Alginate Lys Loaded Nanoparticles

Selected formulations of unloaded and EE/alginate Lys-loaded pNPs were freshly fabricated and evaluated according to terms of size (nm), zeta potential (mV) and PDI (polydispersity). The samples were taken in borosilicate glass vials, filtered using a Syringe Filters 0.22 μm and stored at room temperature (15 to 25 °C) and 37 °C (relative humidity 75%) at time zero, four days, and 1 month using a Malvern NanoZS (Malvern Instruments Ltd., Worcestershire, United Kingdom).

### 2.9. In Vitro Release Test

Freshly fabricated pNPs were separated by centrifugation, lyophilized, and then resuspended in 30 mL of dissolution media (milliQ water). The release study was conducted at 37 °C, under constant agitation and sink conditions, and samples were taken at specific time points for 72 h and quantified by HPLC UV-Vis at 280 nm. The sampling times were: 0.5, 1, 2, 3, 4, 8, 24, 48, and 72 h and 300 µL were withdrawn for Lys quantification. Results were expressed as the percent increase of lysozyme concentration, as follows:

$$\mathrm{Lys}\;\%=\frac{\left[\mathrm{Lysozyme}\right]T_{n}\times 100}{\left[\mathrm{Lysozyme}\right]T_{x}}$$

where [Lysozyme]T_n_ is Lys at sampling times and [Lysozyme]T_x_ is the total lysozyme that can be released.

### 2.10. Cell Culturing and Cell Viability Assays

#### 2.10.1. Models and Cell Culture

Cell viability assays of pNPs were conducted in HeLa cells maintained in DMEM high glucose medium supplemented with 10% FBS, 100 U/mL penicillin, and 100 μg/mL streptomycin in an atmosphere of 95% air and 5% CO_2_ at 37 °C. 

#### 2.10.2. In Vitro Assays

##### MTS Assay

The MTS cellular proliferation assay is routinely used to determine cell viability assessing cell metabolism following nanoparticle exposure. In brief, cells were seeded at a density of 20 × 10^3^ cells/well overnight and treated with the suspensions of vehicle (MiliQ water) and EE/alginate pNPs with negative ZP and positive ZP, diluted in a culture medium at concentrations ranging from 0.06–1.5 μM to determine the effects of vehicle and pNPs on HeLa cell viability. The assay was conducted in 96-well plates in triplicate after 1 and 24 h treatment and then analyzed using a microplate reader (BioTek’s Synergy Mx, Winooski, VT, USA) at 450 and 490 nm. As controls, cells were incubated with equivalent volumes of detergent or vehicle as follows: Death control was 10% SDS (positive control) and vehicle control was MiliQ water (negative control). Cell viability was calculated by comparing the samples to cells incubated with normal culture medium (DMEM/FBS 10%) as 100% survival rate (life control). Cell viability was calculated by comparing the samples to cells incubated with normal culture medium as 100% survival rate.

##### LDH Assay

The Cytotox96 assay from Promega is a colorimetric based cytotoxicity assay that quantitatively measures the release of lactate dehydrogenase (LDH) from damaged cells. The cells were cultured and treated, as described for the MTS assay. After the incubation period, untreated cells of 3 wells were lysed with 10 μL of 10% Triton-X100 in water (Millipore) as a positive control. After 5 min incubation, the plates were centrifuged for 5 min at 400× *g*. The Cytotox96 assay was then performed, as described by the manufacturer and measured at 490 nm. Cell cytotoxicity was calculated by comparing the samples to cells incubated with lysis solution as 100% maximum LDH release.

### 2.11. Morphological Characterization of Interaction of the pNPs with HeLa Cell Lines

#### Cell Morphology

The morphology of the cells incubated with EE/alginate pNPs was observed by Nikon eclipse TE2000U microscope (Tokyo, Japan). The HeLa cells at a density of 20 × 10^3^ cells/mL (seeded in 12-well plates) were treated with the suspensions of vehicle (Mili Q water) and EE/alginate pNPs with negative ZP and positive ZP diluted in culture medium at concentrations ranging from 0.06 to 1.5 μM for 1 and 24 h. After incubation, the cell morphology was observed. The assay was performed in triplicate. Five fields per sample were observed and 30 cells were counted per field to evaluate the possible damages or changes in cell morphology in terms of cell volume increase, roughness, and membrane integrity, among others.

### 2.12. Statistical Analysis

The results were represented as mean ± SD (*n* = 3). Statistical significance of the differences was determined using one-way analysis of variance (ANOVA), followed by the Dunnett or Tukey multiple comparison test at a level of significance of * for *p* < 0.05, ** for *p* < 0.001, and *** for *p* < 0.0001 with GraphPad Prism software (version 4.0; GraphPad software, San Diego, California).

## 3. Results

### 3.1. Nanoparticle Fabrication by Complex Coacervation and Physicochemical Characterization

The method of complex coacervation was successfully used to manufacture the different combinations of unloaded and Lys-loaded EE/alginate nanoparticles according to (Table 1 and Table S1). The nanoparticles exhibited homogenous sizes, as determined using dynamic light scattering (DLS). The pNPs sizes were distributed mainly in a narrow range of 94.1 ± 11.7 and 171.8 ± 4.7 nm for unloaded EE/alginate compared to 112.1 ± 1.1 and 181.5 ± 1.1 nm for Lys loaded EE/alginate nanoparticles. No significant influence of Lys loading on nanoparticle size was observed. Size distribution was narrow, as shown by PDI (from 0.100 to 0.321), indicating homogeneous dispersion for both unloaded and Lys-loaded pNPs.

The zeta potential shows a shift from negative to positive after the precipitation point at 1.0 CR, which is related to the predominance of charged polymers above or below the neutral CR (supplementary data). The PDI results show two types of nanoparticles: The nanoparticles containing predominance of polyanion alginate with CR 0.5 showed negative zeta potential and CR 1.33 and 10 showed that the zeta potential was positive, due to the predominance of polycation EE. Similar results were obtained for unloaded EE/alginate pNPs (Table S1).

Thus, the best results in terms of size and PDI were at CR of 0.5, 1.33 and 10 for TC 20 and 30 (Table 1). Therefore, these formulations were selected to incorporate lysozyme as a model biologic drug and for the following characterization studies.

### 3.2. Scanning Electron Microscopy (SEM)

**Figure 1:**
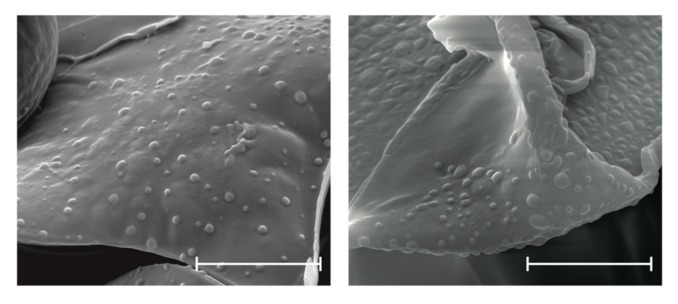
Representative SEM images of (TC 20 CR 0.5) pNPs after the complex coacervation method. Scale bars represent 4 µm.

To determine nanoparticle morphology, SEM studies were conducted. The SEM images revealed the spherical shape of EE/alginate nanoparticles (Figure 1). Interestingly, these particles did not show aggregation or adhesion.

### 3.3. Fourier Transform Infrared Spectroscopy (FT-IR) Study

**Figure 2:**
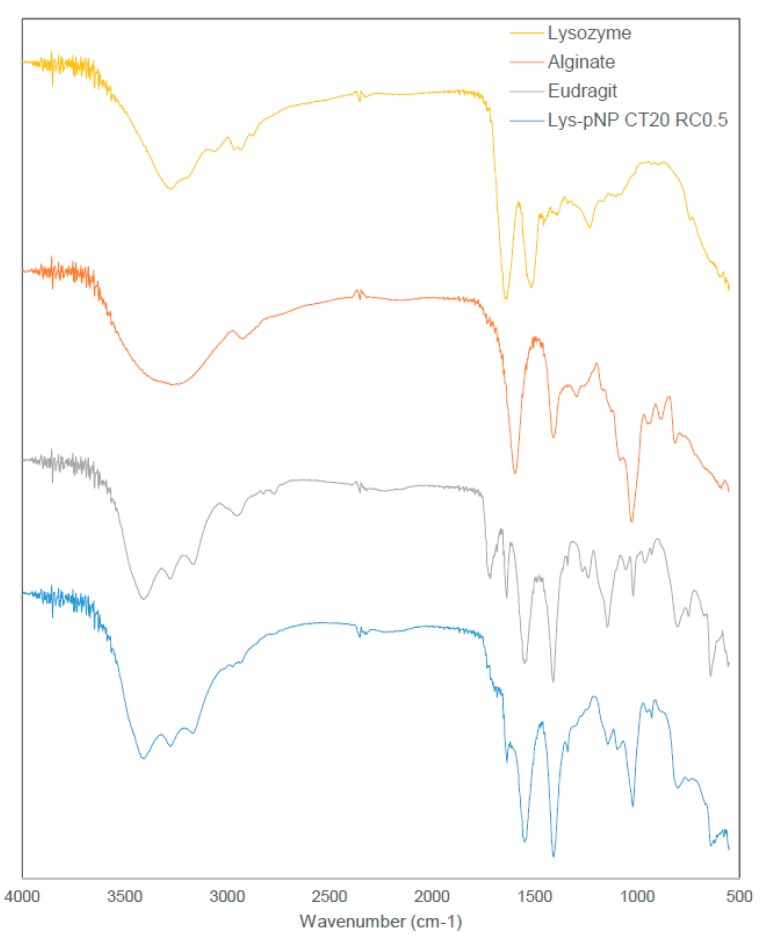
FTIR Spectra of pure materials and Lys-loaded pNP CT 20 and RC 0.5 showing characteristic peaks of each separate material and no evidence of covalent interactions among Lys and materials, supporting the electrostatic interactions that sustain the polymeric system.

Figure 2 shows the FTIR spectra of all pure materials composing pNPs, namely alginate, EE, and Lys exhibiting their respective characteristics peaks. Alginate shows its characteristic peaks at around 3300 cm^−1^ (broad peak for –OH stretching), 1640 cm^−1^ (–COO asymmetric), and 1420 cm^−1^ (–COO symmetric) [45]. The cationic polymer EE shows its characteristics peaks at around 1720 cm^−1^ (for the ester group) and the slight peaks at around 2780 cm^−1^ and 2850 cm^−1^ as evidence of the dimethylamino groups [46,47]. While Lys shows its characteristics peaks at around 1630 cm^−1^ and 1520 cm^−1^ corresponding to its amine bands [48,49,50]. The combined system, exemplified by Lys-loaded pNPs CT 20 and RC 0.5, shows similar peaks to the pure materials with no evidence of new chemical entities. This is aligned with the electrostatic nature of this polymeric system, where no covalent bonds are formed during particle assembly.

### 3.4. Nano Tracking Analysis (NTA)

**Figure 3:**
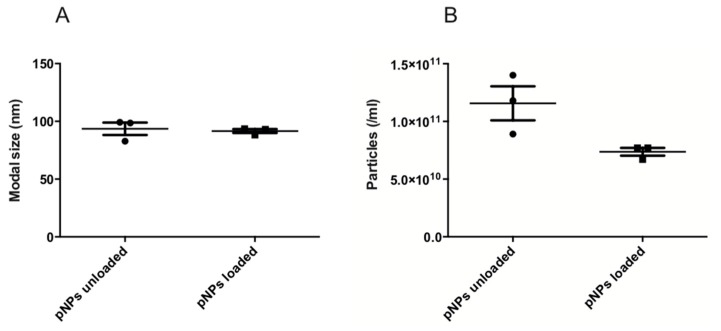
Particle size (**a**) and particles concentration (particles/mL). (**b**) Unloaded EE/alginate pNPs and EE/alginate Lys pNPs (TC 30 CR 1.33 and 30% *w*/*w* Lys). Data represent mean values ± SD (*n* = 3) *p* < 0.05 post hoc *t*-student.

Supplementing the information provided by DLS, NTA enables sample visualization and provides approximate nanoparticles concentrations per mL (Supplementary video). The concentration for unloaded EE/alginate pNPs were 1.1 × 10^11^ particles/mL, while it was 9.0 × 10^11^ particles/mL in the case of Lys loaded EE/alginate pNPs (Figure 3).

Both DLS and NTA showed good sizing accuracy for unloaded EE/alginate (DLS: 106.8 ± 2.5; NTA: 93.5 ± 7.5) and Lys loaded EE/alginate (DLS: 155.1 ± 9.4; NTA: 91.6 ± 2.1) pNPs. Nevertheless, it is possible to observe a tailing of all DLS size distributions towards larger sizes, mostly due to the contribution of a few large particles to the overall scattering (Table 1 and Figure 3). The mean size values obtained by NTA were slightly smaller than data shown by DLS (Table 1). However, the standard deviation of the size distribution obtained for each sample are smaller with DLS (Table 1), which is a consequence of the large amount of statistical data collected by DLS when compared to NTA [51]. In fact, these standard deviations in the NTA results are mostly caused by different particle counts between each measurement (Figure 3) [52].

### 3.5. Lysozyme Association Efficiency

**Figure 4:**
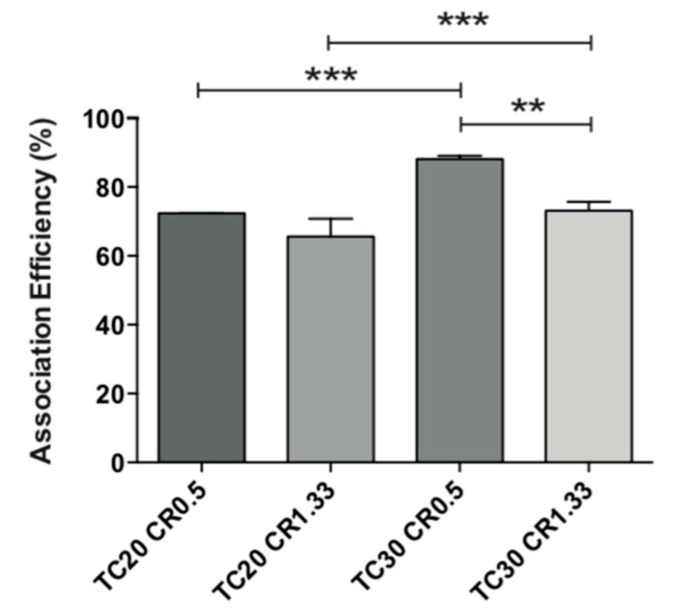
Association efficiency of four different EE/alginate 30% *w*/*w* Lys pNPs with various total charges (TC) and charge ratios (CR). Statistical significant of the differences were assessed by an analysis of variance ANOVA, followed by the Tukey test. ** (*p* < 0.001), *** (*p* < 0.0001) and **** (*p* < 0.00001).

Lys AE% in the tested formulations was high and varied in the range 65–89% (Figure 4). The AE% was always higher for CR 0.5 and independent of the TC. The highest efficiency of association for these nanoparticles corresponds precisely to those with negative ZP; therefore, those with a predominance of the polymer alginate (negative charge) lead to a larger interaction with positively charged lysozyme, thus allowing their greater AE%.

### 3.6. Stability of Lys Loaded EE/Alginate pNPs

**Figure 5:**
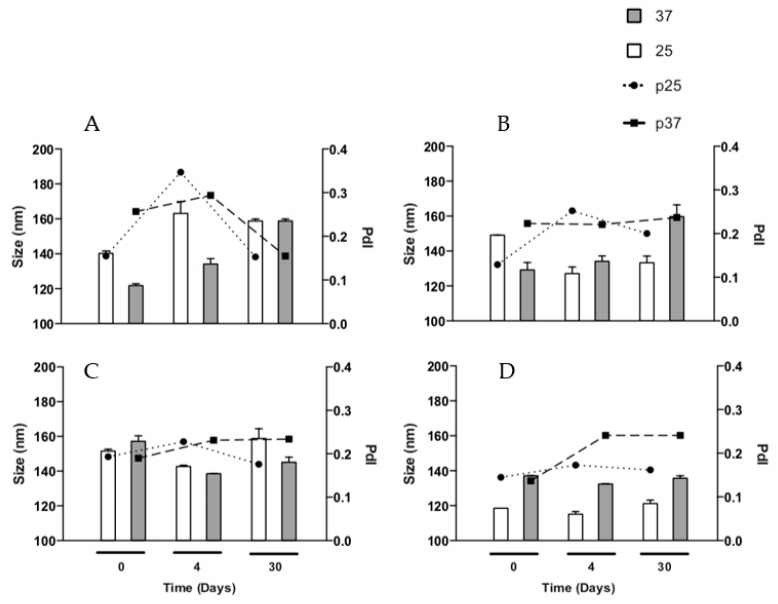
Stability of particle size and PDI of the formulations obtained by DLS at 25 and 37 °C for 0, 4 and 30 days. (**A**) TC 20 CR 0.5; (**B**) TC 20 CR 1.33; (**C**) TC 30 CR 0.5; and (**D**) TC 30 CR 1.33.

The physicochemical properties of Lys loaded EE/alginate pNPs was evaluated after storage for up to 1 month at 25 °C and 37 °C to determine their stability. After 4 and 30 days of incubation at both 25 °C and 37 °C, it was observed that the size of Lys loaded EE/alginate pNPs increased and the PDI at 37 °C remains within acceptable ranges of homogeneous distribution. The pNPs incubated for 4 days at 25°C show the greatest increase in size, which agrees with an increase in polydispersity to a value of 0.34 (Figure 5A). The sizes of Lys loaded EE/alginate pNPs remain stable over the 30 day incubation period for both temperatures evaluated. At an incubation temperature of 37 °C, the PDI of the pNPs remained stable for up to 30 days, while after 4 days of incubation, the PDI of the pNPs incubated at 25° C increased from 0.12 to 0.25 and it reached 0.20 after 30 days (Figure 5B). On the other hand, after 4 and 30 days of incubation, we observed a reduction in size of the Lys loaded pNPs incubated at 37 °C, but the polydispersity is maintained within stable ranges throughout the incubation time and for both temperatures evaluated (Figure 5C).

It is evident that the size of the pNPs incubated at 25 and 37 °C remains stable after 30 days. The polydispersion increases just after 4 and 30 days of incubation for both temperatures (Figure 5D). Overall, these results suggest that the pNPs had a relatively good physical stability during the storage and temperature evaluated. Similar results were obtained for unloaded EE/alginate pNPs (Figure S1).

### 3.7. In Vitro Release Test

**Figure 6:**
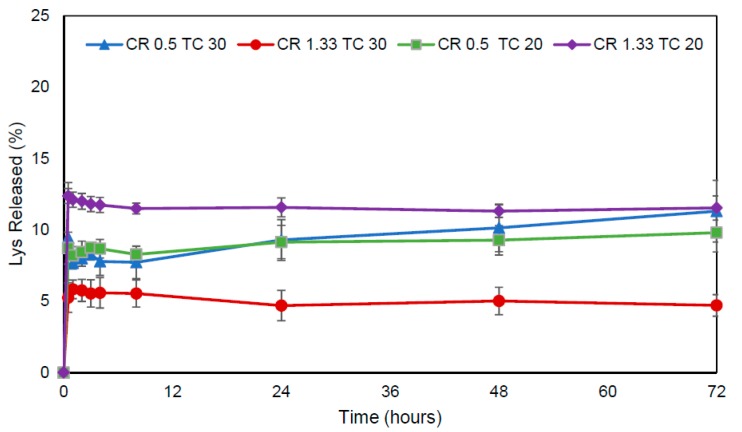
Lys released from pNPs loaded EE/alginate to 30% *w*/*w* in various CR and TC. Data represent mean values ± SD (*n* = 3) *p* < 0.05 post-hoc Tukey’s.

The release profile of all systems was biphasic and characterized by a rapid initial burst (<13%) followed by a sustained lysozyme release (<50%) over a period of 72 h. The initial release kinetics shown by all the evaluated formulations can be attributed to the dissociation of the lysozyme that is physically and loosely bound to the nanoparticle surface, whereas a second and much slower process corresponded to the release of the more tightly associated lysozyme molecules of the EE/alginate nanoparticles as a consequence of a strong electrostatic interaction and a very slow diffusion process out of the polyelectrolyte matrix. Moreover, formulations of pNPs CR 0.5 TC 20 and CR 0.5 TC 30 showed similar release kinetics, possibly due to the high AE% of these formulations and the composition of each nanoparticle (Figure 6).

### 3.8. In Vitro Assays

#### 3.8.1. MTS Assay

**Figure 7:**
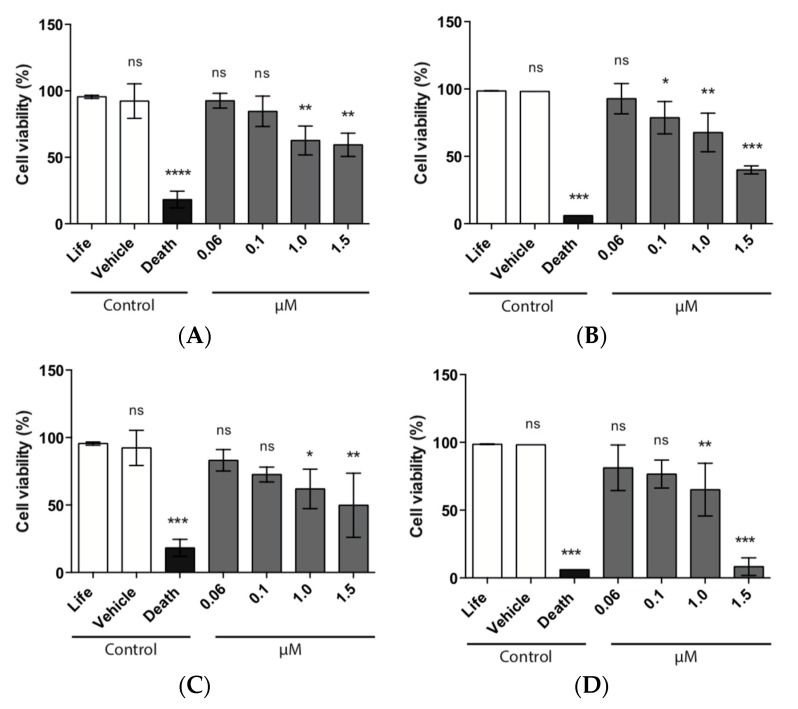
Viability expressed as cell viability percentage of HeLa cells treated with different concentrations of EE/alginate nanoparticles compared to the Culture medium control. (**A**) TC 30 CR 0.5/1 h; (**B**) TC 30 CR 0.5/24 h; (**C**) TC 30 CR 1.33/1 h; and (**D**) TC 30 CR 1.33/24 h.* = *p* < 0.1, ** = *p* < 0.01, *** = *p* < 0.001, ns = no statistically significant difference.

HeLa cells were used to assess the cytocompatibility of the different pNPs tested, using concentrations ranging from 0.06 to 1.5 µM and to elucidate the best non-toxic concentration to be used later. Proliferation of HeLa cells was not affected at the lower concentrations tested of EE/alginate pNPs (0.06 and 0.1 μM). Our results show that cell viability is higher than 90% after exposure to EE/alginate nanoparticles for 24 h. On the other hand, a decrease in cell viability at 1 and 1.5 μM of EE/alginate pNPs, for both positive and negative nanoparticles, was observed after incubation for 1 and 24 h. This was of significance for HeLa cells treated for 24 h with positive nanoparticles (Figure 7).

#### 3.8.2. LDH Assay

**Figure 8:**
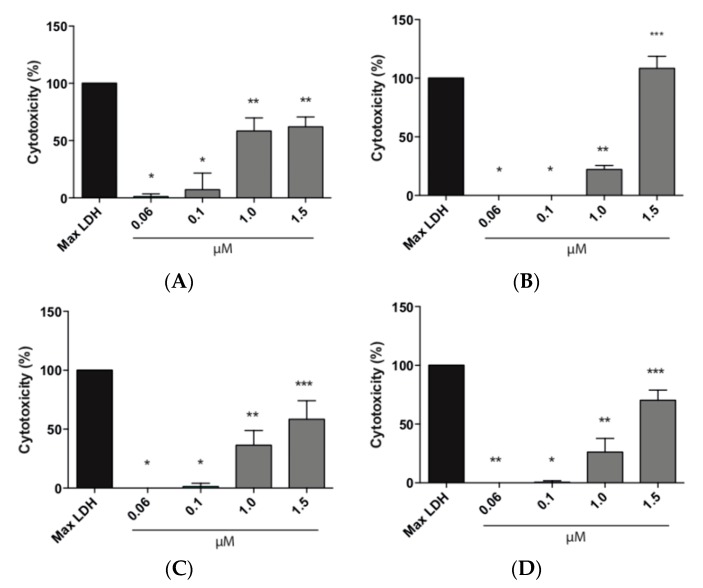
Total LDH release expressed as Cytotoxicity percentage of HeLa cells treated with different concentrations of EE/alginate nanoparticles compared to the Culture medium control. (**A**) TC 30 CR 0.5/1 h; (**B**) TC 30 CR 0.5/24 h; (**C**) TC 30 CR 1.33/1 h; and (**D**) TC 30 CR 1.33/24 h. ** = *p* < 0.01, **** = *p* < 0.0001.

The effect of the EE/alginate nanoparticles on the cell membrane integrity was evaluated by LDH assay that provides an accurate measure of cytotoxicity. Total LDH release was measured from HeLa cells after exposure to different concentrations of EE/alginate nanoparticles. As we can observe in Figure 7, the lower concentrations tested (0.06 and 0.1 µM) had a cytotoxicity percentage ranging between ∼0.0 and 29%. This result shows that these concentrations did not caused any significant damage to the plasma membrane of HeLa cells up to 24 h. On the other hand, HeLa cells treated with higher concentrations (1.0 and 1.5 µM) resulted in a significant increase in LDH release, reaching cytotoxicity values of 50%, and higher as early as after 1 h of incubation. Cytotoxicity levels only reached 100% for negative pNPs after 24h of incubation at the highest concentration (Figure 8).

#### 3.8.3. Cell Morphology

**Figure 9:**
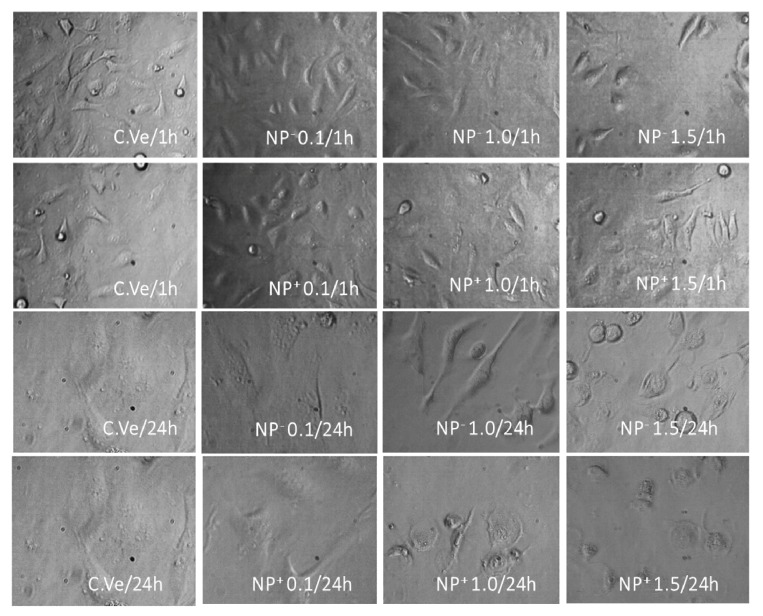
Cell morphology results for various EE/alginate pNPs. HeLa cells were treated with different concentrations (in µmol) of EE/alginate pNPs at either positive or negative zeta potential. C.Ve = Vehicle control. All images taken at 40× magnification.

To study the cell morphological change in response to the EE/alginate pNPs, HeLa cells were incubated with the different preparations or with the vehicle control (C.Ve) corresponding to milli-Q water and observed by phase contrast microscopy. No changes were observed in Vehicle control treated cells in comparison to the normal morphology of the cells. It was also possible to observe normal cell morphology at 0.1 µM of the different EE/alginate pNPs evaluated for both negative pNPs and positive pNPs. On the other hand, our observations showed a slight effect on cell adhesion at higher concentrations of EE/alginate nanoparticles (1.0 and 1.5 μM) with both negative and positive zeta potential. At the same time, only at the higher concentration (1.5 μM) tested after 24 h of incubation could we observe cell constriction and rounding in the cellular morphology of HeLa cells exposed to the treatment (Figure 9).

## 4. Discussion

In this work, we report a new carrier based on complex coacervation of Alg and EE for Lys as a biologic drug model. Our results showed that the nanoformulations varied according to CR and TC of the polymers that participate in the reaction as well as Lys content (% *w*/*v*). All unloaded and Lys loaded nanoparticles resulted in similar average sizes and the ZP was not strongly affected by the presence of lysozyme (Table 1). The ZP values were found to be dependent on the type of polymer that predominates in the formulation. These results are consistent with those of other authors [53].

Most of the nanoparticles were highly stable since most formulations showed ZP > ± 30 mV (and only two formulations with values in the range of ±20–30 mV (Table 1). These data agree with guidelines classifying NP-dispersions with ZP values of ±0–10 mV, ±10–20 mV and ±20–30 mV and ˃±30 mV as highly unstable, relatively stable, moderately stable, and highly stable, respectively [54,55]. Moreover, the PDI values, which provides information about the homogeneity of particle size, was below 0.35 in all formulations.

The characterization of the nano-formulations using SEM showed the characteristic spherical morphology of the EE/alginate pNPs formed. FTIR spectra did not show evidence of covalent interactions among constituent materials and also suggested the absence of Lys denaturation. Electrostatically sustained polyelectrolytes do not rely on new covalent bonding contributing to the matrix structure, and as such, FTIR spectra has been used in the past to show that functional groups remain identifiable in combined systems [56]. FTIR spectra also suggests that Lys secondary structure is retained after pNP synthesis observed in the peak at around 1630 cm^−1^ as opposed to changes that could be observed during Lys denaturation [57,58].

To characterize size, DLS analysis showed a size 106.8 nm and a PDI of 0.221 for the EE/alginate unloaded pNPs, indicating a moderate poly-dispersity sample. This was confirmed by particle visualization in the NTA. Furthermore, the mean size obtained by NTA was 93.5 nm, is about 13.3 nm smaller than the size given by DLS, indicating a certain degree of polydispersity. This can be explained by the fact that size distributions obtained by DLS are intensity distributions, whereas NTA provides number distributions, which results in a higher polydispersity. Another explanation for the polydispersity index may be due to the inherent difficulty for DLS to properly analyze polydisperse samples [59]. The same phenomenon can explain the results obtained for EE/alginate Lys loaded nanoparticles.

Lysozyme was incorporated into EE/alginate pNPs with high association efficiency (65–89%), which can correlate to the amphoteric nature of Lyz, a glycoprotein, that forms complexes with positively and negatively charged compounds, which favor association efficiency [60]. Moreover, the high association efficiency for formulations with negative zeta potential can be attributed to the predominance of alginate in these formulations. As such, Alg reduces the loss of Lys due to its intrinsic flexibility capacity that allows it to accommodate lysozyme molecules, generating a dense matrix of interaction phenomenon, as described by Fuenzalida et al. and Saha et al. [24,61].

The release of lysozyme from pNPs EE-alginate involves a ‘burst effect’ during the first stage of drug release, where 12.4%, 9.4%, 8.7%, and 5.2% of Lys was released from pNPs within the first 30 min. The initial burst release was attributed to the surface-bound lysozyme in the nanoparticle formulation [62]. After which the release was very slow and the release profile of the four nanoparticle formulations were similar after 72 h. These observations provided clear evidence of the capacity of pNPs to modulate the lysozyme release profile (Figure 6). Interestingly, the release study showed that smaller pNPs in size TC 20 CR 1.33 were the ones in which the fastest burst effect was observed, followed by pNPs TC 30 CR 0.5, which corresponds to the second smaller formulation showing this phenomenon. This is in accordance with what was has been described by Agnihotri et al. [63]. Moreover, the remaining load after the burst release could potentially be released intracellularly for the biologics pharmacological effect, as previously described [63].

Using different approaches, we demonstrated that the decrease in cell viability of HeLa cells treated with EE/alginate nanoparticles was dose-dependent, as the treatment with higher concentrations (1.0 and 1.5 μM) of EE/alginate pNPs inhibited the proliferation HeLa cells ranging from 67.7% to 8.3% (Figure 6). On the other hand, no significant decrease in cell viability was observed at the lower concentrations tested (0.06 and 0.1 μM) up to 24 h, both with positive and negative pNPs. Cytotoxicity of EE/alginate nanoparticles was also determined, and no significant increase of cytotoxicity levels was observed for pNP concentrations ranging from 0.06 to 0.1 μM. On the other hand, at the higher pNPs concentrations evaluated, the cytotoxicity levels reached significant levels as early after 1 h of incubation. The level of cytotoxicity obtained using these concentrations of EE/alginate pNPs is similar to what is usually observed for these types of treatments using other cellular models [64,65,66].

According to our MTS and LDH results, in all cases the cytotoxicity of EE/alginate nanoparticles was dose-dependent, and we observed the same behavior dependent on the surface charge of the nanoparticle, as described above (Figure 7 and Figure 8), as we observed that positive nanoparticles were more cytotoxic to HeLa cells in all conditions tested. This result can be attributed to the differences on cell pNPs interactions. Other studies have shown that highly positively charged pNPs are more likely to have higher interactions with the cell membrane leading to higher cytotoxicity [67,68,69]. As both types of pNPs were obtained using the same polymer and had similar sizes, the difference in zeta potentials is the factor that accounts for their differential interaction forces. Therefore, it is possible that the stronger interaction forces resulted in higher cytotoxicity from positively charged pNPs.

In accordance with our previous results, the direct observation of treated and control HeLa cells reveals that cell morphology was only affected at the highest concentration tested (1.5 μM), after 24 h of exposure for both positive and negatively charged pNPs. In these cases, we observed constriction and rounding in cellular morphology of HeLa cells exposed to the treatment. This reduction in cell volume corresponds to a single event that occurs in cell death by apoptosis and allows differentiation of cell death by necrosis characterized by the loss of membrane integrity and irreversible cytoplasm swelling. It has been described that charged nanoparticles induce cell death by apoptosis, while neutral nanoparticles induce necrosis [68].

HeLa cells, a human epithelial carcinoma cell line, have been used to assess the toxicity of different types of nanoparticles, although results are conflicting. This is due to various reasons, including the way results are analyzed and shown. Most notably, it is difficult to determine a consensus in nanoparticles concentration and its effect in cell viability and morphology [70,71]. Thus, the in vitro nanoparticle dose needs to be assessed individually for each formulation. Moreover, the comparative toxicity of nanoparticles cannot be determined without a clear understanding of the physical and physicochemical characteristics of these materials [72]. In this work, we used different physiological concentrations and several experimental approaches to assess their cytocompatibility in HeLa cells.

## 5. Conclusions

We were able to generate a new carrier based on complex coacervation of alginate and a cationic polymethacrylate (EE) for delivery of the model enzyme lysozyme. By controlling TC and CR, we have shown that it is possible to control the size and surface properties resulting in pNPs that have a moderate polydispersity and can incorporate Lys efficiently. Moreover, their cytocompatibility characterization showed promising results in cell viability and cytotoxicity with only the higher concentrations of pNPs having a damaging effect in HeLa cells. Overall, our results show the potential of these novel Eudragit EE/alginate polymeric nanoparticles for their use in biomedical applications and can lead the way in future coacervation nanocarrier development for biologics drug delivery.

## Figures and Tables

**Table 1:** Characteristics of the formulation, in terms of size, PDI, and zeta potential (*n* = 3). Results are expressed as means ± standard deviations.

Formulation		Size (nm)		PDI		ZP (mV)	
TC	CR	EE/Alginate Unloaded	EE/Alginate lyz Loaded	EE/Alginate Unloaded	EE/Alginate lyz Loaded	EE/Alginate Unloaded	EE/Alginate lyz Loaded
10	0.5	110.4 ± 1.6	112.1±1.1	0.100 ± 0.002	0.224 ± 0.016	−40.1 ± 0.1	−50.4 ± 5.0
10	1.33	130.3 ± 18.7	161.3 ± 67.8	0.144 ± 0.075	0.321 ± 0.035	39.3 ± 1.2	50.6 ± 3.7
10	10	122.7 ± 1.7	121.1 ± 1.8	0.114 ± 0.027	0.160 ± 0.009	44.0 ± 3.1	25.5 ± 1.2
20	0.5	171.8 ± 4.7	181.5 ± 1.1	0.153 ± 0.130	0.193 ± 0.016	−37.3 ± 1.2	−30.6 ± 0.4
20	1.33	132.2 ± 0.6	118.5 ± 0.0	0.150 ± 0.004	0.145 ± 0.001	27.6 ± 3.6	35.3 ± 0.0
20	10	94.1 ± 11.7	133.9 ± 1.2	0.233 ± 0.046	0.212 ± 0.006	44.7 ± 4.7	43.9 ± 0.2
30	0.5	154.5 ± 5.7	158.8 ± 1.1	0.176 ± 0.019	0.191 ± 0.044	−42.4 ± 1.2	−40.0 ± 1.0
30	1.33	106.8 ± 2.5	155.1 ± 9.4	0.221 ± 0.012	0.154 ± 0.016	38.1 ± 1.6	38.1 ± 0.9
30	10	150.4 ± 3.2	158.8 ± 3.4	0.181 ± 0.10	0.262 ± 0.001	38.5 ± 0.8	33.7 ± 2.0

## Supplementary Materials

The following are available online at https://www.mdpi.com/1999-4923/11/3/103/s1, Figure S1. Particle size and PDI of the Lys unloaded formulations obtained by DLS at 25 and 37 °C for 0, 4 and 30 days. A: TC 30 CR 1.33; B: TC 30 CR 10. Table S1. Characteristics of the Lys unloaded formulation, in terms of size, PDI and zeta potential (*n* = 3).
