# Supplementary material for: Development of Novel EE/Alginate Polyelectrolyte Complex Nanoparticles for Lysozyme Delivery: Physicochemical Properties and In Vitro Safety
Source: Pharmaceutics. 2019 Mar 1;11(3):103. doi: 10.3390/pharmaceutics11030103 (PMC6470925; doi:10.3390/pharmaceutics11030103)
Supplement: Supplementary file 1 [file pharmaceutics-11-00103-s001.pdf]

pharmaceutics

MDPI

Supplementary Materials: Development of Novel EE/Alginate Polyelectrolyte Complex Nanoparticles for Lysozyme Delivery: Physicochemical Properties and in Vitro Safety

Sabrina Sepúlveda-Rivas, Hans F. Fritz, Camila Valenzuela, Carlos A. Santiviago and Javier O. Morales

The following supplementary materials are available online: Figure S1: Particle size and PDI of the Lys unloaded formulations obtained by DLS at 25 and 37 °C for 0, 4 and 30 days. A: TC 30 CR 1.33; B: TC 30 CR 10. Table A1: Characteristics of the Lys unloaded formulation, in terms of size, PDI and zeta potential (*n* = 3). Video A1: EE/alginate pNPs visualization.

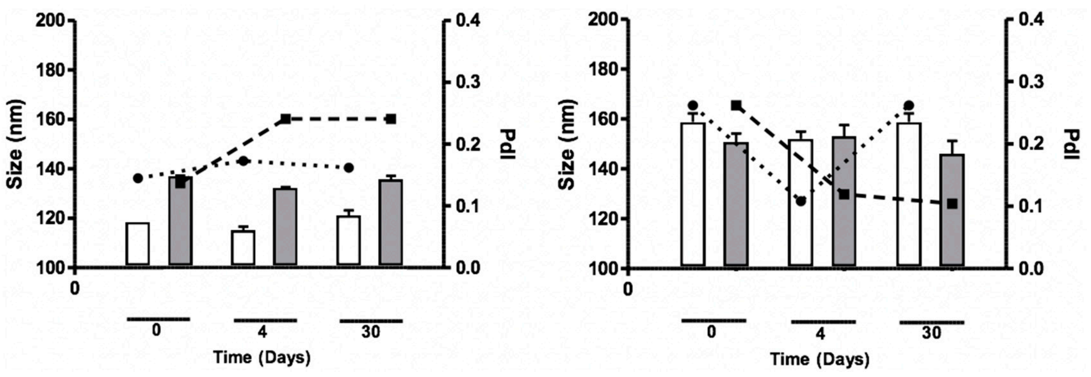

Figure S1. Particle size and PDI of the Lys unloaded formulations obtained by DLS at 25 and 37 °C for 0, 4 and 30 days. A: TC 30 CR 1.33; B: TC 30 CR 10.

Table S1. Characteristics of the Lys unloaded formulation, in terms of size, PDI and zeta potential (*n* = 3).

Formulation		Size (nm)	PDI	Zeta Potential (mV)
TC (n+ + n-)	CR (n+/n-)			
2	0.1	83.8 ± 4.6	0.356 ± 0.017	−28.93 ± 0.0
2	0.25	91.4 ± 4.9	0.294 ± 0.040	−14.87 ± 3.1
2	0.5	101.1 ± 0.7	0.132 ± 0.005	−17.47 ± 1.2
2	0.75	71.4 ± 0.7	0.335 ± 0.012	41.80 ± 4.6
2	1	61.45 ± 1.8	0.417 ± 0.033	39.53 ± 8.6
2	1.33	87.36 ± 6.6	0.519 ± 0.104	48.27 ± 7.0
2	2	120.57 ± 1.1	0.658 ± 0.136	39.40 ± 11.0
2	4	167.33 ± 7.4	0.655 ± 0.117	51.00 ± 1.9
2	10	256.70 ± 23.0	0.542 ± 0.097	50.03 ± 3.8
4.4	0.1	101.32 ± 1.4	0.265 ± 0.007	−36.83 ± 2.4
4.4	0.25	113.23 ± 1.5	0.176 ± 0.015	−27.97 ± 1.2
4.4	0.5	145.17 ± 1.7	0.136 ± 0.023	16.07 ± 0.6
4.4	0.75	78.85 ± 1.0	0.334 ± 0.030	26.10 ± 1.8
4.4	1	67.70 ± 0.8	0.405 ± 0.040	30.57 ± 0.6
4.4	1.33	121.03 ± 8.7	0.417 ± 0.150	28.27 ± 2.4
4.4	2	131.39 ± 42.5	0.498 ± 0.206	27.30 ± 5.0
4.4	4	174.87 ± 34.0	0.3650.170	27.20 ± 7.6
4.4	10	238.80 ± 35.1	0.596 ± 0.155	28.30 ± 3.6

6.0	0.1	107.60 ± 1.1	0.219 ± 0.010	−27.00 ± 3.9
6.0	0.25	117.37 ± 1.2	0.174 ± 0.017	−22.57 ± 5.2
6.0	0.5	135.57 ± 1.17	0.161 ± 0.003	24.43 ± 1.5
6.0	0.75	79.57 ± 1.08	0.203 ± 0.015	24.17 ± 2.9
6.0	1	pp	pp	pp
6.0	1.33	65.73 ± 1.49	0.213 ± 0.015	58.53 ± 5.0
6.0	2	56.65 ± 1.34	0.236 ± 0.008	32.33 ± 1.3
6.0	4	72.37 ± 3.64	0.371 ± 0.018	36.40 ± 2.3
6.0	10	112.60 ± 1.87	0.357 ± 0.025	45.03 ± 4.4
